# Application of Fractal Analysis in Detecting Trabecular Bone Changes in Periapical Radiograph of Patients with Periodontitis

**DOI:** 10.1155/2021/3221448

**Published:** 2021-10-07

**Authors:** Parisa Soltani, Sajad Sami, Jaber Yaghini, Ehsan Golkar, Francesco Riccitiello, Gianrico Spagnuolo

**Affiliations:** ^1^Department of Oral and Maxillofacial Radiology, Dental Implants Research Center, Dental Research Institute, School of Dentistry, Isfahan University of Medical Sciences, Isfahan, Iran; ^2^Students' Research Committee, School of Dentistry, Isfahan University of Medical Sciences, Isfahan, Iran; ^3^Department of Periodontics, Dental Implants Research Center, Dental Research Institute, School of Dentistry, Isfahan University of Medical Sciences, Isfahan, Iran; ^4^Medical Image and Signal Processing Research Center, School of Advanced Technologies in Medicine, Isfahan University of Medical Sciences, Isfahan, Iran; ^5^Department of Neurosciences, Reproductive and Odontostomatological Sciences, University of Naples “Federico II”, Naples, Italy; ^6^Institute of Dentistry, I. M. Sechenov First Moscow State Medical University, Moscow 119435, Russia

## Abstract

**Introduction:**

Evaluation of detailed features of the supporting bone is an important step in diagnosis and treatment planning for teeth with clinical attachment loss. Fractal analysis can be used as a method for evaluating the complexity of trabecular bone structures. The aim of this study was to evaluate the trabecular bone changes in periapical radiographs of patients with different stages of periodontitis using fractal analysis.

**Methods:**

This comparative cross-sectional study was performed on patients with and without clinical attachment loss in mandibular first molars. Teeth with clinical attachment loss were divided into mild, moderate, and severe periodontitis groups. Digital periapical radiographs were obtained from the mandibular first molars using the same exposure parameters. DICOM file of the radiographs was exported to ImageJ software for fractal analysis. Three regions of interest (ROIs) were considered in each radiograph: two proximal ROIs mesial and distal to the mandibular first molar and one apical ROI. Fractal dimension (FD) values were calculated using the fractal box counting approach. Statistical analysis was performed using the chi-square test, Mann–Whitney test, intraclass correlation coefficient, and ANOVA (*α* = 0.05).

**Results:**

FD values were significantly different between moderate and severe periodontitis and healthy periodontal bone (*P* < 0.05), except for the distal ROI for moderate periodontitis cases (*P*=0.280). However, FD values of the supporting bone in periodontally healthy teeth and teeth with mild periodontitis did not show a statistically significant difference (*P* > 0.05).

**Conclusion:**

Fractal analysis is a useful tool for evaluation of bone alterations in moderate and severe periodontitis, but was not able to detect the most initial radiographic bone signs of mild periodontitis.

## 1. Introduction

Periodontal disease is one of the most important causes of tooth loss and can be considered as a modifying factor in many systemic diseases [1–4]. Alveolar bone changes can indicate onset and progression of periodontal diseases. Therefore, assessment of changes in the alveolar bone structure can be an indispensable measure in prevention, treatment planning, and prognosis of periodontal diseases. Radiography is an important tool used in different phases of periodontal evaluation, and different imaging modalities can be used to monitor the status of periodontal tissues, including panoramic radiography, intraoral radiography, and cone beam computed tomography [5–7]. However, bone changes can be visible in radiographs only if more than 30% of the mineral content of the bone has undergone resorption [8]. Therefore, additional analysis of radiographs could potentially enhance the diagnostic application of these images for detection of details.

Fractal analysis is an adjunct method for quantitative evaluation of bone trabeculation [9, 10]. This technique detects complex structural patterns in the trabecular bone and quantitatively determines the complexity of the bone using a measure called fractal dimension (FD). Calculated FD in periapical radiograph demonstrates the complexity of the alveolar bone structure surrounding the teeth [11]. Among the existing methods for calculating FD, the box-counting approach explained by White and Rudolph is mostly used for binary images such as periapical radiographs. In this method, FD is basically a measure of the number of boxes needed to cover the trabecular pattern. Higher FD shows that the trabecular pattern is more complex [12].

A number of studies have been performed on evaluation of trabecular changes in the alveolar bone in patients with periodontitis [10, 13–15]. However, none of these studies have separately assessed mild, moderate, and severe periodontitis. Therefore, the aim of the present study was to evaluate the trabecular bone changes in periapical radiographs of patients with different stages of periodontitis using fractal analysis.

## 2. Materials and Methods

### 2.1. Patient Selection and Clinical Examination

This comparative cross-sectional study was approved by the Ethical Committee of Isfahan University of Medical Sciences (^#^IR.MUI.RESEARCH.REC.1399.538). This study was performed on patients requiring periapical radiographs of vital mandibular first molars. Inclusion criteria were patients not taking any drugs or supplements, without systemic disorders, history of trauma, or congenital deformities, as well as vital teeth without endodontic lesions, endodontic treatments, and restorative crowns. Patients were excluded if they were not willing to participate in the study or if their radiographs had any artifact or bone lesion in the region of interest. A sample size of 36 patients in each group was required for demonstrating a difference of 0.092 in FD between the groups, with alpha of 0.05 and power of 80%.

Periodontal assessment was performed by one operator using an intraoral mirror and a periodontal probe. Patients with a clinical attachment loss of 1-2 mm were recorded as mild, 3-4 mm as moderate, and more than 4 mm as severe periodontitis. In addition, individuals without clinical attachment loss will be selected as the healthy group [16].

### 2.2. Radiographic Examination and Analysis

Digital periapical radiographs were obtained using the paralleling technique by size 2 imaging plates (Durr Dental, Bietigheim-Bissingen, Germany) by standard posterior film holders (Kerr, California, USA). All radiographs were taken by an intraoral X-ray tube (Planmeca, Helsinki, Finland) with exposure parameters of 70 kVp, 8 mA, and 0.16 s.

Bone level in the mesial and distal regions of the first molar was measured on radiographs in Scanora software (Soredex, Helsinki, Finland) from the cementoenamel junction to the alveolar crest.

**Figure 1:**
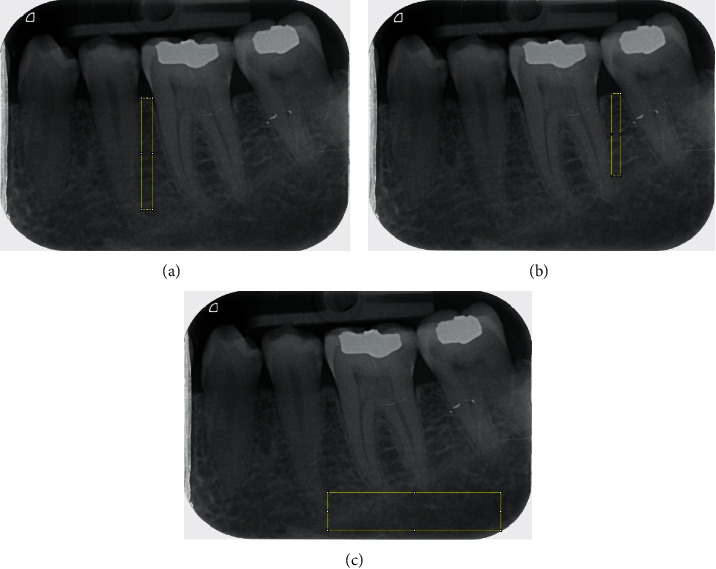
Selected regions of interest (ROIs) in periapical images: (a) mesial ROI, (b) distal ROI, and (c) apical ROI.

For fractal analysis, DICOM file of the radiographs was exported to ImageJ (NIH, USA). In each radiograph, three regions of interest (ROIs) were considered: the apical ROI was selected as the biggest rectangle extending horizontally from mesial of the mandibular first molar to the most posterior molar or the posterior border of the image and vertically from molar apexes to the inferior border of the image or the inferior alveolar canal. Proximal ROIs were selected in the mesial and distal regions as rectangles extending from the alveolar crest to the line connecting the apex of the first molar and its adjacent teeth (Figure 1).

Selected ROIs were cut out of the original image using “Clear Outside” tool. The resultant regions were then duplicated, and “Gaussian Blur” filter with a sigma of 10 was applied on the second image in order to reduce noise. Thereafter, the filtered image was subtracted from the original image, and “Make Binary” tool was used to produce a black and white image. Mean intensity was set at 128 (8 bit image), and then, “Fractal Box Count” tool was used to calculate FD. Image analysis was performed by a trained senior dental student. 10 radiographs were analyzed after one month in order to determine intraobserver agreement.

### 2.3. Statistical Analysis

Quantitative data were presented as median values (IQR). The distribution of data was analyzed using the Kolmogorov–Smirnov test. Statistical analysis was performed by Statistical Package for the Social Sciences (SPSS, v. 25, IBM, NY, USA) using the chi-square test, Mann–Whitney test, intraclass correlation coefficient, and ANOVA (*α* = 0.05).

## 3. Results

Periapical radiographs were taken from 36 patients in the healthy group (14 males and 22 females) and 44 patients in the periodontitis group (17 males and 27 females; 18 mild, 13 moderate, and 13 severe). Excellent intraobserver agreement (ICC = 0.96, *P* < 0.001) was found for determination of FD in different ROIs. Demographic information of the study participants is given in Table 1. Sex distribution was not statistically different between the periodontitis and healthy groups (*P*=0.98). However, patients with periodontitis were significantly older than the healthy population (*P* < 0.001). FD values were significantly different between moderate and severe periodontitis and healthy periodontal bone (*P* < 0.05), except for the distal ROI for moderate periodontitis cases (*P*=0.280). However, FD values of the supporting bone in periodontally healthy teeth and teeth with mild periodontitis did not show a statistically significant difference (*P* > 0.05) (Table 2).

## 4. Discussion

The findings of the present study indicated that fractal analysis can be used to distinguish between the periodontal bone in healthy teeth and teeth with moderate and severe clinical attachment loss. In periapical radiographs, FD was lower in the surrounding bone of teeth with periodontitis compared with those without attachment loss. This study is the first one to attempt to detect subtle bone changes caused as a result of mild periodontitis using fractal analysis.

Incorporating enhanced analysis on medical images is an important step for several diagnostic tasks. A minimum change of 30% is required for visual detection of differences in bone structures in radiographs [8, 17]. However, the use of fractal analysis, which is based on mathematical morphology for analysis of the bone trabecula, is a method which can theoretically enhance the diagnostic potential of radiographs [14]. In fact, the findings of the present study indicated that fractal analysis could detect the bone alterations of moderate and severe periodontitis. Several studies have been performed using fractal analysis as a tool for evaluation of bone structure for different conditions and purposes, ranging from evaluation of dental implants [18, 19], temporomandibular disorders [20], and bruxism [21] to effects of celiac disease [22] and lactation [23] on mandibular bone structure. The present study was an attempt to incorporate fractal analysis in detection of periodontitis which is a common dental condition. Since periapical radiographs are routinely used combined with clinical examination for diagnosis, treatment planning, and prognosis evaluation of teeth with periodontal involvement, an ample opportunity exists for fractal analysis to provide an added value to conventional radiographs.

A number of previous studies have been performed on application of fractal analysis in detection of periodontitis based on radiographs. One of the first studies on the subject was the study performed by Shrout et al. in which they reported that in the posterior mandibular region, the FD values of the interdental bone are higher in patients with gingivitis compared with patients with periodontitis [13]. Updike and Nowzari in their study on 108 radiographs of mandibular anterior teeth with healthy periodontium, moderate periodontitis, and severe periodontitis concluded that FD values were higher in the healthy periodontal bone [10]. A study performed by Cha et al. performed on healthy teeth and teeth with furcation involvement revealed that FD values in the proximal region are not different between the control and furcation involvement group, while FD values in the furcation region are significantly different between the two patient populations [24]. The findings of the study performed by Cha et al. are not in line with our findings. However, in their study, furcation involvement was the criterion for selection of teeth with periodontitis which was not considered in the inclusion criteria of the present study. In addition, Grassl and Schulze have used digitized film radiographs for their analysis. The process of digitization results in reduction of the dynamic range of radiographs lead to loss of radiographic information [25]. In the present study, digital radiographs were used for fractal analysis allowing for a wider dynamic range and thus more accurate results. Sener et al. have also successfully incorporated fractal analysis for differentiating moderate and severe periodontitis from healthy periodontal status in periapical radiographs [14]. Aktuna Belgin and Serindere in 2020 showed FD values in the mesial and distal regions that are significantly higher in the periodontitis group compared with the healthy group [6]. However, as mentioned, our study was the first one to consider patients with mild periodontal attachment loss among the study participants. Additionally, in the present study, proximal and apical ROIs were considered in the periapical images.

One limitation of the present study was that for standardization purposes, all periapical radiographs were taken from the mandibular first molars. The present findings can be investigated in different locations of the jaws to see if regional differences affect the diagnostic potential of fractal analysis.

## 5. Conclusion

Fractal analysis is a useful tool for evaluation of bone alterations in moderate and severe periodontitis, but was not able to detect the most initial radiographic bone signs of mild periodontitis.

## Figures and Tables

**Table 1:** Demographic information of study participants.

	Number	Male (%)	Female (%)	Mean age (SD)
Healthy	36	14 (38.9)	22 (61.1)	23.31 (7.25)
Periodontitis	44	17 (38.6)	27 (61.4)	36.95 (11.24)
Total	80	31 (38.8)	49 (61.3)	30.81 (11.87)

**Table 2:** Median (IQR) values of fractal dimension in different regions of interest in study groups.

Fractal dimension	Healthy	Periodontitis			*P* value
		Mild	Moderate	Severe	
Apical	1.66 (1.50–1.69)	1.62 (1.54–1.67)	1.52 (1.38–1.62)	1.32 (1.24–1.55)	Healthy vs. mild (0.650)
					Healthy vs. moderate (0.043)^*∗*^
					Healthy vs. severe (<0.001)^*∗*^

Mesial	1.63 (1.59–1.66)	1.60 (1.56–1.64)	1.55 (1.43–1.64)	1.54 (1.42–1.58)	Healthy vs. mild (0.740)
					Healthy vs. moderate (0.035)^*∗*^
					Healthy vs. severe (0.001)^*∗*^

Distal	1.60 (1.55–1.63)	1.57 (1.51–1.61)	1.58 (1.53–1.59)	1.42 (1.36–1.56)	Healthy vs. mild (0.210)
					Healthy vs. moderate (0.280)
					Healthy vs. severe (<0.001)^*∗*^

^*∗*^Statistical significance.

## Data Availability

The data used to support the findings of this study are included within the article.
